# Probing the hair detectability of prohibited substances in sports: an in vivo-in silico-clinical approach and analytical implications compared with plasma, urine, and faeces

**DOI:** 10.1007/s00204-023-03667-1

**Published:** 2024-01-15

**Authors:** Shao-Hsin Hung, Hung-Lin Kan, Chun-Wei Tung, Yi-Ching Lin, Ting-Ting Chen, Ciao Tian, William Chih-Wei Chang

**Affiliations:** 1 Doctoral Degree Program in Toxicology, College of Pharmacy, Kaohsiung Medical University, Kaohsiung, 807 Taiwan; 2 Institute of Biotechnology and Pharmaceutical Research, National Health Research Institutes, Miaoli County, 350 Taiwan; 3 Department of Laboratory Medicine, School of Medicine, College of Medicine, Kaohsiung Medical University, Kaohsiung, 807 Taiwan; 4 Department of Leisure Industry and Health Promotion, College of Humanities and Management, National Ilan University, Yilan County, 260 Taiwan; 5 School of Pharmacy, College of Pharmacy, Kaohsiung Medical University, Kaohsiung, 807 Taiwan

**Keywords:** Anti-doping, Sports drug testing, Anabolic steroids, Diuretics, Alternative matrix, World Anti-Doping Agency

## Abstract

Hair analysis is a crucial method in forensic toxicology with potential applications in revealing doping histories in sports. Despite its widespread use, knowledge about detectable substances in hair is limited. This study systematically assessed the detectability of prohibited substances in sports using a multifaceted approach. Initially, an animal model received a subset of 17 model drugs to compare dose dependencies and detection windows across different matrices. Subsequently, hair incorporation data from the animal experiment were extrapolated to all substances on the World Anti-Doping Agency’s List through in-silico prediction. The detectability of substances in hair was further validated in a proof-of-concept human study involving the consumption of diuretics and masking agents. Semi-quantitative analysis of substances in specimens was performed using ultra-performance liquid chromatography–tandem mass spectrometry. Results showed plasma had optimal dose dependencies with limited detection windows, while urine, faeces, and hair exhibited a reasonable relationship with the administered dose. Notably, hair displayed the highest detection probability (14 out of 17) for compounds, including anabolic agents, hormones, and diuretics, with beta-2 agonists undetected. Diuretics such as furosemide, canrenone, and hydrochlorothiazide showed the highest hair incorporation. Authentic human hair confirmed diuretic detectability, and their use duration was determined via segmental analysis. Noteworthy is the first-time reporting of canrenone in human hair. Anabolic agents were expected in hair, whereas undetectable compounds, such as peptide hormones and beta-2 agonists, were likely due to large molecular mass or high polarity. This study enhances understanding of hair analysis in doping investigations, providing insights into substance detectability.

**Supplementary Information:**

The online version contains supplementary material available at 10.1007/s00204-023-03667-1.

## Introduction

Human sports doping worldwide is controlled in reference to an annually-published list of prohibited substances by the World Anti-Doping Agency (WADA) (World Anti-Doping Agency 2023). The List encompasses over 300 substances, divided into three categories: prohibited at all times, prohibited in-competition, and prohibited in particular sports. Samples collected from sports competitors, including urine and blood, are routinely screened for the presence of these substances, primarily using mass spectrometry. Technological innovations have led to substantial improvements in today’s strategies for sports drug testing. These innovations encompass longitudinal monitoring of biological variables through the Athlete Biological Passport, the discovery of new long-term metabolites, and target testing triggered by intelligence (Thevis et al. 2019). Nonetheless, distinguishing between chronic intentional doping scenarios and acute inadvertent drug exposures based on a single test of conventional biofluids remains challenging. The question of ‘when’ a doping agent was administered is particularly difficult to address. Thus, additional supporting information is warranted to enhance the result management process in doping control.

While blood provides a ‘minutes to hours’ readout of the drug's metabolic state, urine offers a ‘hours to days’ pattern of excreted drug metabolites (Caplan and Goldberger 2001). Hair has been featured as a valuable analytical specimen for the long-term and retrospective detection of drugs (Thevis et al. 2016). Compared to short-lived drug presence in biofluids, hair analysis measures over ‘months to years’, correlating with specific exposure times due to hair’s average growth rate of about one centimetre per month (Kintz 2007). Substances can be deposited in hair after administration through blood circulation, involving three primary mechanisms: (1) transportation from blood capillaries into growing cells located between the matrix cells and the end of the keratinisation zone of the hair follicle, (2) transfer from deep skin compartments during hair shaft formation, and (3) introduction via sweat or sebum secretions into the hair shaft (Kamata et al. 2015; Pragst and Balikova 2006).

Hair testing has been commonly employed in the field of forensic toxicology, particularly when investigating cases of poisoning or death involving exposure to substances of abuse, including amphetamines, cannabinoids, cocaine, and opiates (Ferreira et al. 2019). Its potential application extends to unveiling the history of doping in sports. However, the knowledge of which substances are detectable in hair is still limited to a handful of selected compounds identified from very few real cases, encompassing both post-mortem scenarios (Gheddar et al. 2021; Kintz et al. 2019, 2021a) and instances involving athletes (Favretto et al. 2019; Gheddar et al. 2020; Kintz et al. 2020a). The true incorporation of substances into hair is achieved through their absorption and distribution within an organism, a phenomenon that cannot be accurately replicated by artificially spiking or soaking hundreds of chemical standards onto hair shafts, as has been noted elsewhere (Wong et al. 2018). Distinct disparities in the mechanisms of drug distribution between soaked hair samples and actual hair from users have been established through imaging observations on hair sections (Kamata et al. 2020).

To enhance our understanding of detectable doping substances in hair, this study introduced an animal model of receiving a subset of model compounds that represent specific classes of substances prohibited in sports, as administering them one by one to an organism would be impractical. The investigation centred on the comparisons of the substance levels found among the biospecimens (e.g., hair, urine, blood, and faeces) that were derived from an assured amount of substance exposure to the system, thereby disregarding potential drug–drug interactions within the mixture. This study aims to: (1) compare dose dependencies and detection windows for substances among different matrices, (2) calculate degrees of hair incorporation using experimental data and extrapolate to all substances on the List via in-silico prediction, and (3) validate hair detectability through a proof-of-concept human study involving chronic diuretics consumption.

## Methods

### Drug mixture for oral gavage

The standards, chemicals, and reagents used in this study are listed in Text S1. A mixture of selected drugs is prepared for administration to rats via oral gavage. The selection of drugs was based on several considerations. Firstly, the substances prohibited at all times (in- and out-of-competition) in sports from the Prohibited List (World Anti-Doping Agency 2023) were chosen. Secondly, the substances with higher Adverse Analytical Finding (AAF) occurrence rates in each drug class from the Anti-Doping Testing Figures Report (World Anti-Doping Agency 2021) were prioritized. Thirdly, the oral bioavailability of each substance and the accessibility of its standard from the manufacturer were assessed. In total, 17 substances from 4 classes were selected as the model drugs which encompassed S1 anabolic agents (stanozolol, methyltestosterone, testosterone, and clenbuterol), S3 beta-2 agonists (terbutaline and salbutamol), S4 hormone and metabolic modulators (tamoxifen, clomifene, anastrozole, GW1516, letrozole, and trimetazidine), and S5 diuretics and masking agents (furosemide, hydrochlorothiazide, canrenone, chlorothiazide, and probenecid).

The given doses of the drugs were determined from a compromise between the considerations of drug toxicity and analytical sensitivity. After reviewing the LD_50_ of each drug, anastrozole has the lowest oral LD_50_ of > 100 mg/kg in rats (Pfizer 2011). An amount 50 times lower than this LD_50_ dose was chosen for each drug to minimise the potential adverse effects on animals. Respectively, 0.5 × dose was set at 1 mg/kg, 1 × dose at 2 mg/kg, and 2 × dose at 4 mg/kg for oral gavage in rats of this study. The retrieved toxicological information for each drug is summarised in Table S1.

From the preliminary tests, to prepare the mixture solution, drugs with log*P* smaller than 3.25 were dissolved using dimethyl sulfoxide, and drugs with log*P* larger than 3.25 were dissolved using corn oil. The log*P* of each drug was calculated using a log*P* predictor (Grob 2022) (Table S1). Then, two solutions were mixed and diluted with corn oil to the final concentrations for gavage in 1% dimethyl sulfoxide in corn oil (*v*/*v*). Proper sonication was performed to ensure all drugs were completely dissolved. The mixture solution was prepared 2 days before the administration and was stored at 4 °C. The volume of oral gavage in rats was 5 mL/kg.

### Animal treatment

**Fig. 1:**
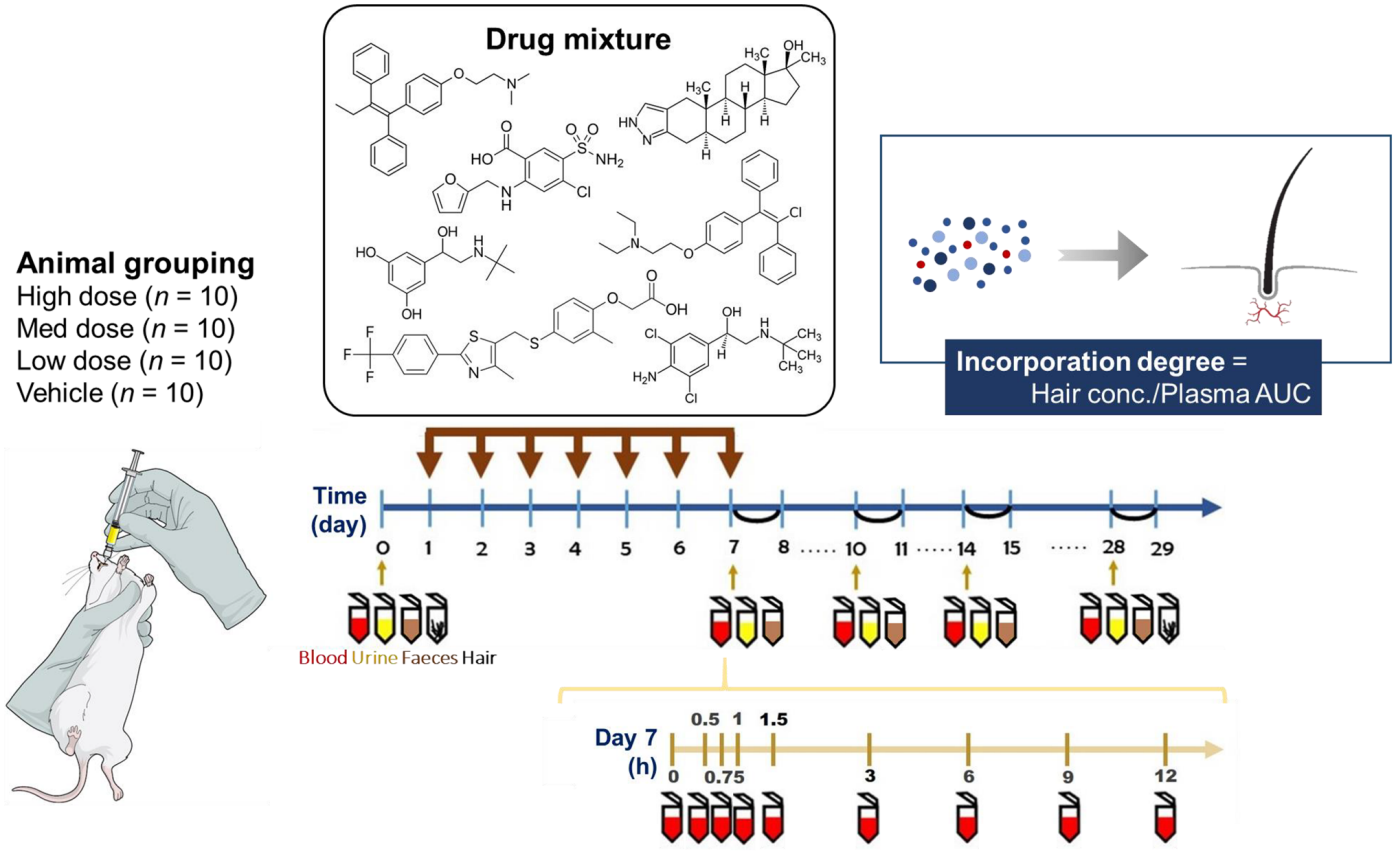
Experimental design for animal treatment

The protocol for animal treatment was reviewed and approved by the Institutional Animal Care and Use Committee of Kaohsiung Medical University (Approval No.: 110139). Six-week-old male rats were purchased from BioLASCO (Taipei, Taiwan) and kept in regularly cleaned plastic cages, with a maximum of 4 animals per cage. Food (Altromin International, Lage, Germany) and pure water were provided ad libitum. The experimental design is shown in Fig. 1. After a 2-week acclimation period, 40 rats were randomly assigned to four groups: vehicle (*n* = 10), 0.5 ×-dose (*n* = 10), 1 ×-dose (*n* = 10), and 2 ×-dose (*n* = 10). The ‘cocktail’ of prohibited substances dissolved in corn oil was administered by oral gavage once every 24 h for a period of 7 days.

The hair specimens from the rat dorsal skin were shaved and collected on days 0 and 28. The 24-h urine and faecal specimens were collected on days 0, 7, 10, 14, and 28 using individually-housed metabolic cages. The blood specimens were collected from the tail vein on days 0, 7, 10, 14, and 28. Particularly on day 7, additional blood was withdrawn at 0.5, 0.75, 1, 1.5, 3, 6, 9, and 12 h after the drug administration to obtain the area under the plasma drug concentration–time curve (AUC). All specimens were stored at − 80 °C until analysis.

### Sample preparation

When analyzing a wide range of physiochemical properties of drugs from very different specimens, the sample preparation necessitates harmonisation given achieving a meaningful comparison of their levels between hair, plasma, urine, and faeces. The consideration of hair samples was prioritized as its preparation is the most complex among all. Based on the preliminary optimisation (Text S2) in conjunction with our previous experience (Chang et al. 2020) and available publications (Cooper et al. 2012; Vogliardi et al. 2015), the first step for hair sample preparation is the decontamination phase to remove possible external contamination. Hair and faeces are solid matrices and thus require an efficient homogenisation process. On the other hand, enzymatic hydrolysis of glucuronide-conjugated drugs is adopted for urine preparation. After the addition of internal standards, each sample can then enter the extraction process. Protein precipitation with acetonitrile was chosen for sample extraction as it provides a wider coverage for molecules (but lower selectivity). A uniform extraction method for all matrices, by mixing homogenised samples or biofluids with equal volume of solvent, can yield as equivalent recoveries as possible. The final volumes of extract were also set to be the same. The detail of preparation procedures for four matrices are given in Text S3.

### Semi-quantitation

For each matrix, a matrix-matched calibration curve was prepared at 15 concentration levels within the range of 0.01–500 pg/mg for hair and faeces (ng/mL for plasma and urine), by fortifying the standard mixture of 17 analytes before the sample preparation. The amount of standard solutions in the samples was no more than 5% to avoid the dilution effects of matrix components. The ratios of analyte-to-internal standard responses were used for semi-quantitation to compensate for extraction recoveries, matrix effects, and systematic errors. Testosterone-d3 was used for correcting the analytes of S1.1 anabolic agents; clenbuterol-d9 for S1.2 other anabolic agents; terbutaline-d9 for S3. beta-2 agonists; tamoxifen-d5 for S4. hormone and metabolic modulators; furosemide-d5 for S5. diuretics and other masking agents. The range of the calibration curve for semi-quantitation of each analyte was obtained from the lowest concentration of the estimated limit of detection (LOD)—having a signal-to-noise (S/N) ratio ≥ 3 in the extracted ion chromatogram—to the highest concentration of 500 pg/mg (ng/mL). The calibration curve was constructed using at least six levels, and the concentration of test samples was estimated through extrapolation if they fell outside the linear range. In the analytical sequences, a procedural blank and a quality control sample were run in parallel with the test samples.

### Ultra-performance liquid chromatography–tandem mass spectrometry

Analyses were performed on an Acquity UPLC I-Class Plus system coupled to a Xevo TQ-S micro mass spectrometer (Waters, Milford, MA, USA). Chromatographic separation was performed on an ACQUITY UPLC BEH C18 2.1 × 100 mm column (i.d. 1.7 μm). Mobile phases consisted of water (A) and acetonitrile:methanol (8:2, *v*/*v*) (B), both containing 0.1% formic acid, by applying the following gradient program: 0–10 min, 5–95% B; 10–11 min, 95% B; 11–11.5 min, 95–5% B; 11.5–13 min, 5% B. Other chromatographic conditions were as follows: column temperature, 40 °C; sample temperature, 10 °C; injection volume, 2 μL; and flow rate, 0.45 mL/min. Mass spectrometry detection was performed under positive and negative ionisation in one run, using the multiple reaction monitoring (MRM) mode. General MS parameters were as follows: capillary voltage, 2.5 kV; desolvation temperature, 350 °C; desolvation gas, 650 L/h. The optimised MRM transitions, cone voltages, collision energies, and retention times for the analytes were listed in Table S2. MassLynx and TargetLynx software (Waters, Milford, MA, USA) were used for data acquisition and processing, respectively.

### Hair incorporation: experimental calculation and in silico prediction

The hair incorporation degree of each substance was calculated using the hair concentration divided by the plasma AUC (Nakahara and Kikura 1996). This ratio indicates the tendency of a substance to be incorporated from the system (blood) into the hair. The collected data from the rat experiment were then extrapolated to all prohibited substances on the WADA’s List via in-silico prediction.

**Fig. 2:**
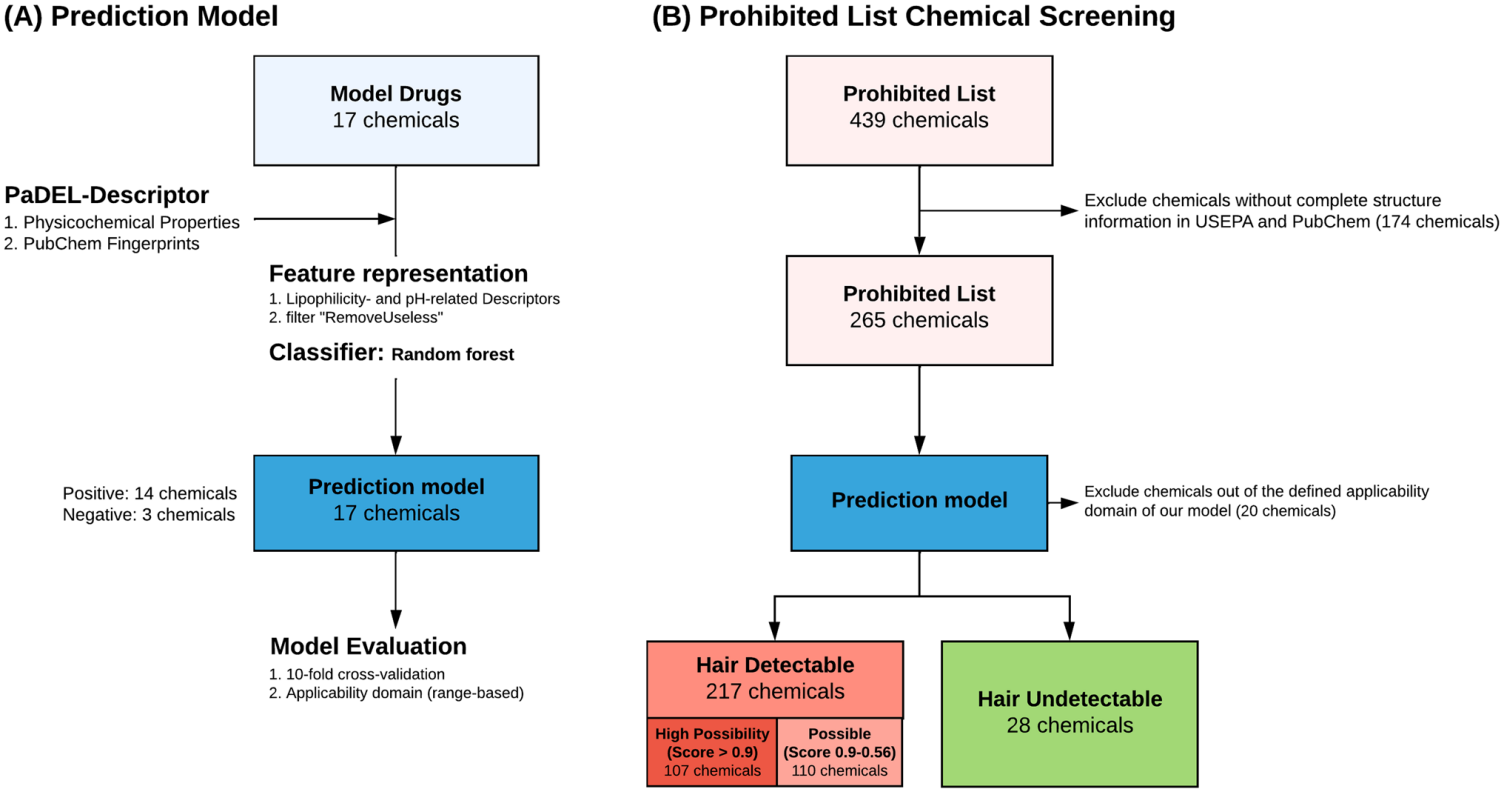
General workflow of in silico prediction for hair incorporation. **A** Quantitative structure–activity relationship model development and **B** screening result for substances on the prohibited list

The general workflow of quantitative structure–activity relationship (QSAR) model development is briefly explained herein (Fig. 2A). The substances utilized in this QSAR study included both positive chemicals (can be detected in hair) and negative chemicals (can only be detected in urine). Datasets were prepared from a series of hair incorporation results and calculated parameters of chemical structures from PaDEL-Descriptor (Yap 2011).

Previous studies have proven that the hair incorporation of substances is evidently affected by the substances’ physicochemical properties, particularly lipophilicity, basicity, and molecule mass (Nakahara and Kikura 1996; Pragst and Balikova 2006). Therefore, feature representation is performed by collecting lipophilicity and pH-related descriptors, and removing irrelevant features with a variance percentage higher than 99% by the filter "RemoveUseless" in WEKA (version 3.8.3). The QSAR model was constructed using Random Forest (RF) (Breiman 2001). The tenfold cross-validation method and range-based applicability domain were applied for evaluating our QSAR model to avoid overestimation of its performance. Finally, a set of 265 substances, with available structure information from the United States Environmental Protection Agency (USEPA), in the Prohibited List were applied in the computational screening of hair incorporation possibility.

### Proof-of-concept human study

The protocol of human hair collection was reviewed and approved by the National Cheng Kung University Human Research Ethics Committee (Approval No.: NCKU HREC-E-109-449-2). All participants signed informed consent forms, and the study was conducted in accordance with the Declaration of Helsinki and approved guidelines. Volunteers who are known to have taken one of the model drugs, particularly diuretics and masking agents, which is of great interest and their detectability has not been well-validated after reviewing available literature, were recruited in this proof-of-concept study. The volunteers were recruited from hospitals and local pharmacies. The experimental design involved the collection of authentic hair specimens from participants, who, due to personal health considerations, had been prescribed the drugs under investigation. A questionnaire was administered upon collection to document relevant information, including the dosage of medication and the condition of the hair, such as whether there were any instances of dyeing or perming. The hair collection method involved grasping hair near the scalp at the occipital region, cutting the hair close to the roots with scissors, and securing the location near the scalp with tape for identification. Upon collection, the hair samples were carefully stored and transported to the laboratory. Subsequently, the collected hair samples were then cut into 2-cm segments. The analytical steps, which encompassed hair decontamination, sample preparation, and analysis, followed the methods employed in animal experimentation, ensuring consistency across the study procedures.

## Results

### Analytical method for semi-quantitation

**Fig. 3:**
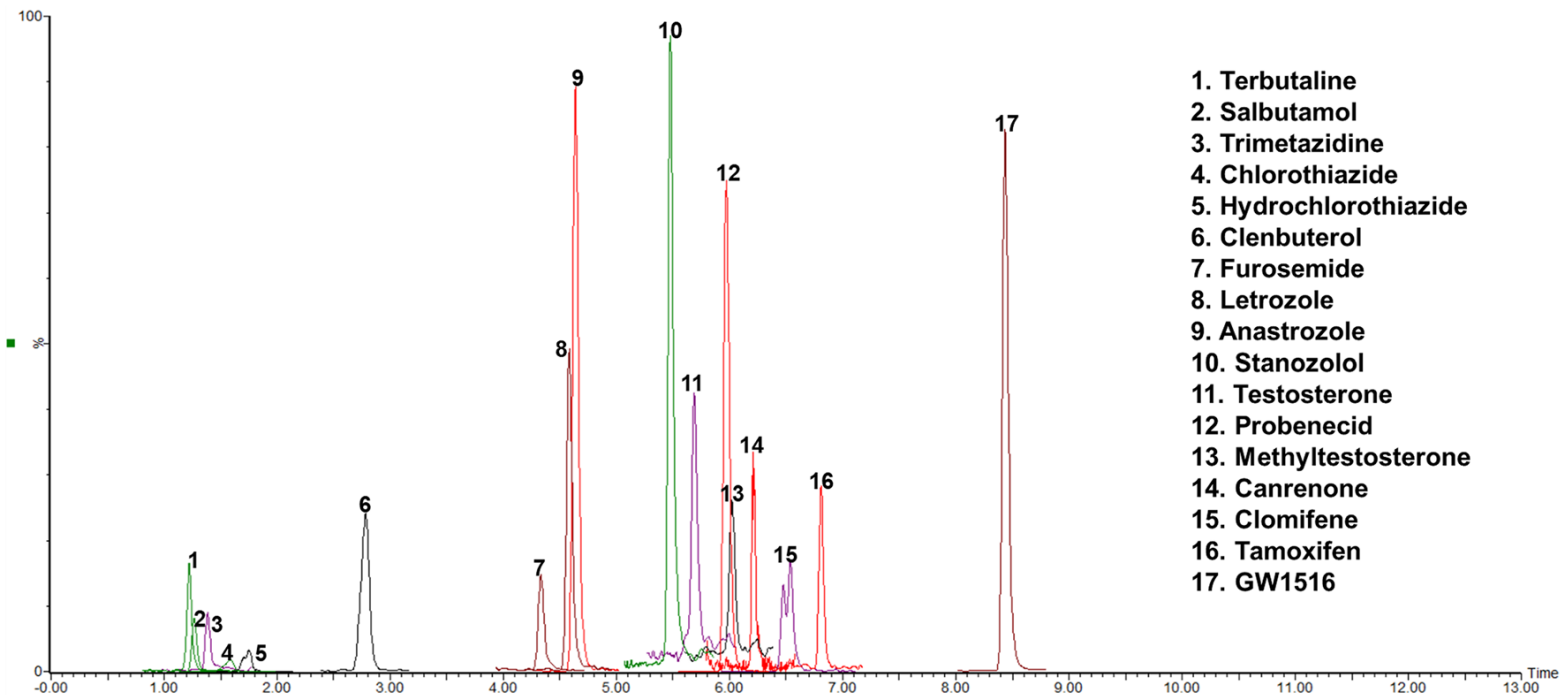
Extract ion chromatogram of the quantifying ions of analytes in hair samples analyzed by LC–MS/MS

The extraction chromatograms of the hair samples fortified with reference standards are depicted in Fig. 3. The matrix-matched calibration curves for each specimen showed acceptable linearity, with the correlation coefficient (*r*^2^) exceeding 0.99 for most of the analytes. The lowest points on the calibration curves ranged from 0.01 to 10 pg/mg for solid matrices (ng/mL for liquid matrices). Hair and faecal specimens generally exhibited greater complexity in their blank matrices, as evident in the chromatogram; therefore, for the most part, higher detection limits for hair and faeces can be observed compared to plasma and urine (Table S3).

### Dose dependencies and detection windows for substances among matrices

**Fig. 4:**
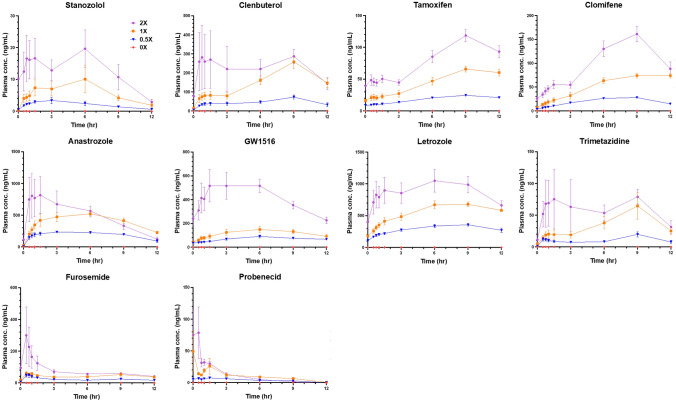
Concentration–time curves estimated for substances in rat plasma on the 7th day after the last administration. Values are shown as the mean ± SEM for *n* = 10 rats

**Fig. 5:**
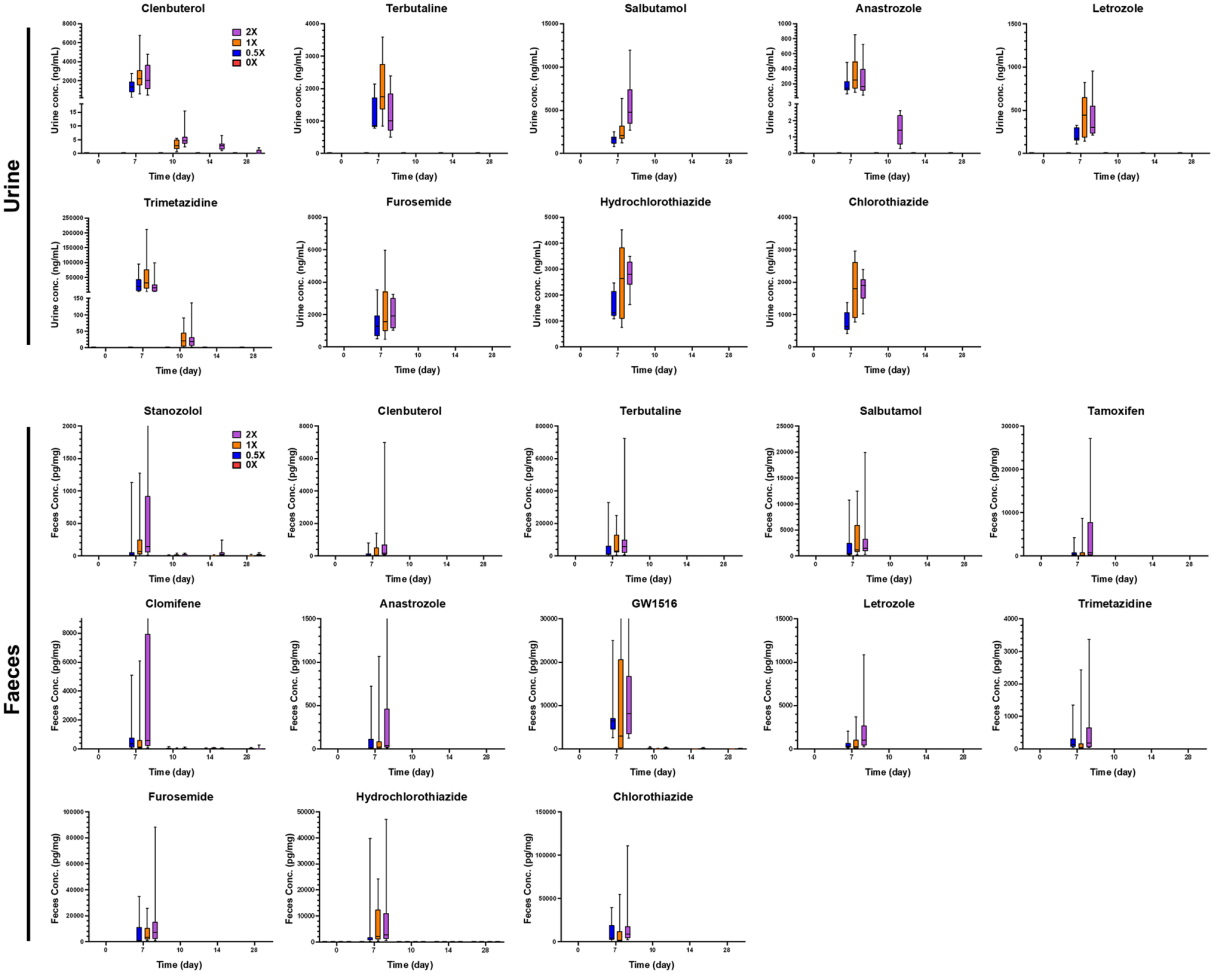
Estimated concentrations for substances in rat urine and faeces throughout the study period. Values are shown as the mean ± SEM for *n* = 10 rats

The maximum concentrations for each substance between doses in each matrix are given in Table S4. Plasma concentrations and AUCs showed the best correlation to the given doses for the majority of the substances (Fig. 4). Urinary and faecal concentrations generally displayed a reasonable relationship with the given doses, although larger variations were clearly observed, resulting in disparities in dose dependencies within certain substances (Fig. 5). Hair concentrations also demonstrated robust dose dependencies among the three doses in most substances, particularly the hormone and metabolic modulators. Blunted responses were observed in stanazolol, methyltestosterone, hydrochlorothiazide, canrenone, and probenecid, especially when comparing 0.5 × and 1 ×.

In plasma, the majority of the substances remained detectable for merely 12 h after the last administration (Fig. 4). They were not detected at the next time point, 3 days after the last administration. Clenbuterol, GW1516, and letrozole had extended detection windows, remaining detectable for 7 days, 14 days, and 3 days after the last administration, respectively. Most substances in urine and faeces were detected on the last day of administration (day 7). In urine, clenbuterol exhibited the longest detection period, lasting until day 28, while anastrozole and trimetazidine had detection period of day 10. In faeces, stanazolol, clomifene, and GW1516 displayed extended detection windows, reaching up to day 28 (Fig. 5). Hair samples were collected on day 28 of the experiment, which was 21 days after the last administration.

### Hair incorporation: experimental

**Fig. 6:**
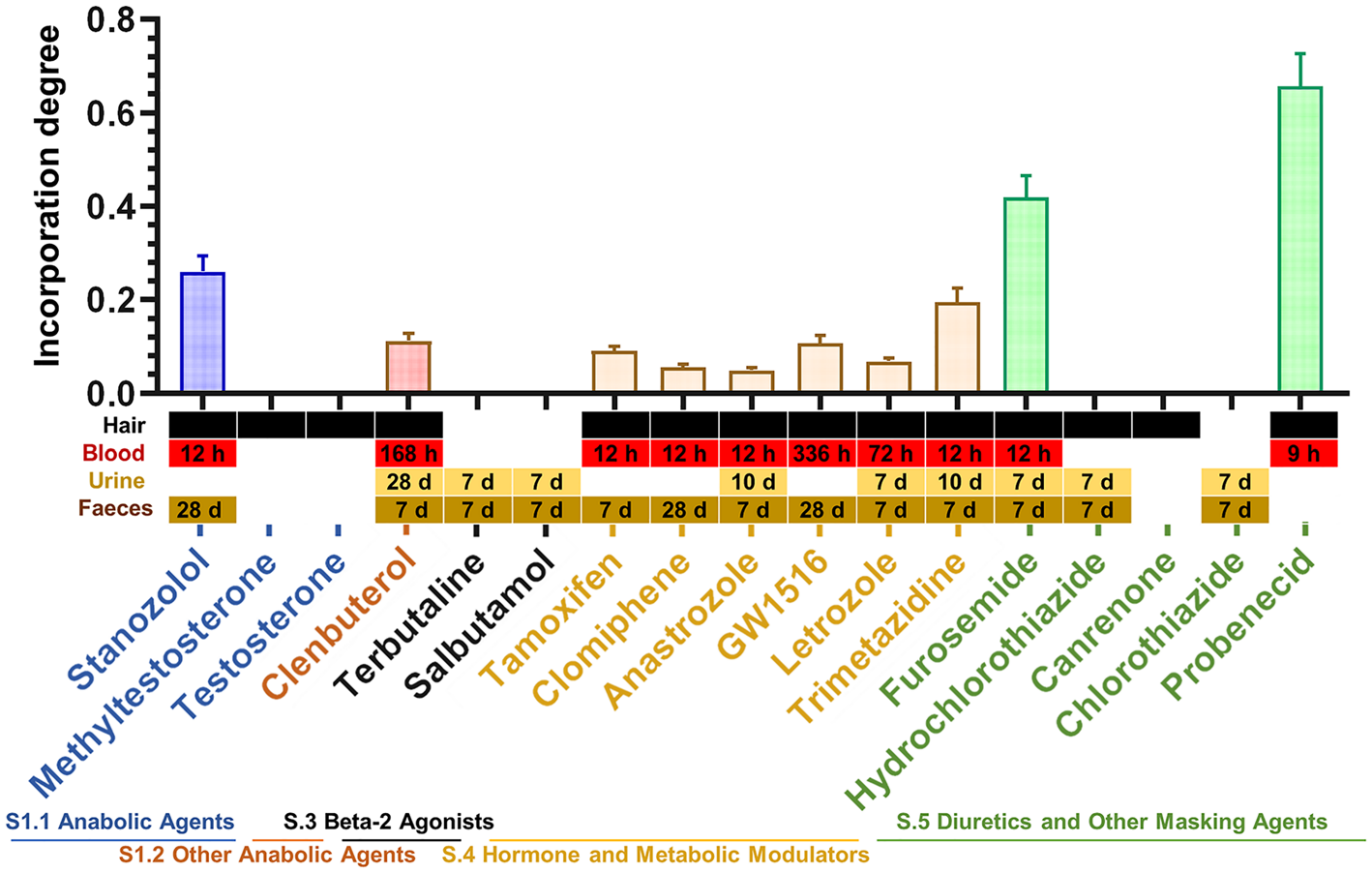
Hair incorporation degrees of substances in rats, calculated by dividing hair concentration by plasma AUC. Values are shown as the mean ± SEM for 30 rats. The grid below the column chart presents the detection windows of each substance between specimens

Out of the 17 model drugs, 14 were detectable in hair, 10 in blood, 9 in urine, and 13 in faeces (Fig. 6). The incorporation degree could only be determined when the substance was detected in both hair and plasma. Therefore, incorporation degrees were calculated for 10 substances, since methyltestosterone, testosterone, hydrochlorothiazide, and canrenone were detected in hair but not in plasma. Among the anabolic agents, the mean incorporation degrees for stanozolol and clenbuterol were 0.26 and 0.11, respectively. The beta-2 agonists, terbutaline, and salbutamol were not detected in the hair. All six of the studied hormone and metabolic modulators were well incorporated into the hair, with mean incorporation degrees ranging from 0.05 to 0.20. The diuretics displayed the highest levels, specifically furosemide with a degree of 0.42 and probenecid with a degree of 0.66. Hydrochlorothiazide and canrenone were both detected in the hair but not in the blood, while chlorothiazide was not detected in either hair or blood.

### Hair incorporation: in silico

A total of 17 model drugs were utilized to construct a prediction model based on the RF algorithm and exhibited 100% accuracy in distinguishing between positive and negative substances within our training dataset. To comprehensively evaluate the Prohibited List, we collected the chemical structure information from USEPA and PubChem, and a total of 265 substances were obtained for further evaluation (Fig. 2B). Among these, we identified 20 substances that were not in the AD as defined by our prediction model and were not included in further analysis.

**Fig. 7:**
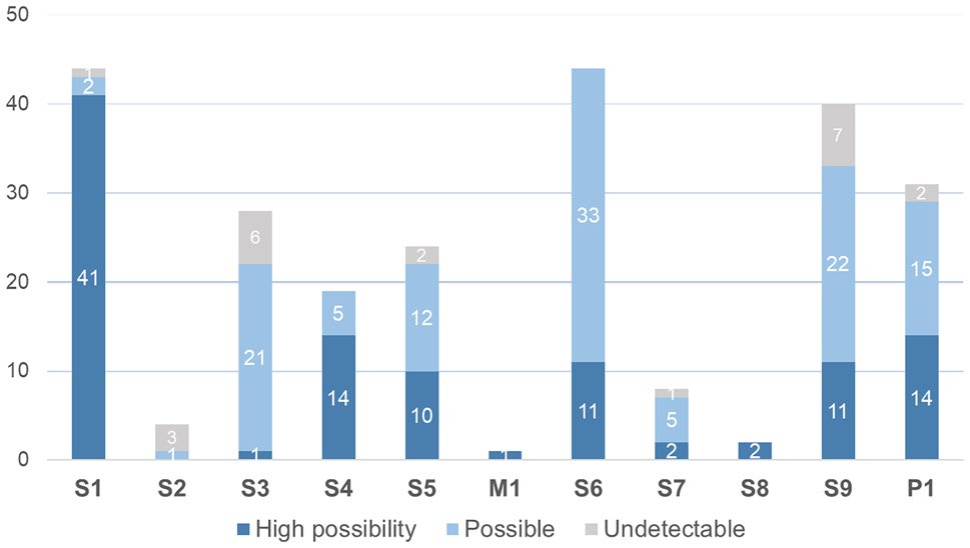
Quantities and distribution of the 245 substances on prediction for hair detectability

Of the 245 prohibited substances, our model predicted 217 substances to be detectable in hair and the remaining 28 were classified as undetectable. Notably, a subset of 107 substances displayed a high score over 0.9, indicating a high confidence of predicted presence. The full list of prediction result is provided in Table S5. The substances with high scores showed a predominant focus on S1 anabolic agents, encompassing a total of 41 substances. Conversely, the 28 substances predicted undetectable in hair covered 7 classes, and most of them were categorized as S2 peptide hormones and growth factors, S3 beta-2 agonists, and S9 glucocorticoids (Fig. 7).

### Diuretics and masking agents in human hair

Human hair samples were collected from one patient who received letrozole, two patients who received furosemide, one who received furosemide, canrenone, and hydrochlorothiazide, one who received furosemide and canrenone, one who received canrenone, and two who received hydrochlorothiazide. In segmental hair analysis of subject F1, whose hair was permed and dyed, the concentrations of letrozole decreased from the proximal to distal segments. The letrozole concentrations were determined as follows: 1608 pg/mg in the 0–2 cm segment, 950 pg/mg in the 2–4 cm segment, 507 pg/mg in the 4–6 cm segment, and 121–268 pg/mg between 6 and 24 cm (Table 1). Diuretics furosemide, canrenone, and hydrochlorothiazide were all confirmed to be detectable in authentic human hair; however, drugs were not detected in subjects with a drug use duration of less than 5 weeks (i.e., subjects F3 and M1). In addition, the drug use duration is correlated with its distribution in the hair strand. Subject F6, who received 5-week hydrochlorothiazide, had a concentration of 93 pg/mg in the 0–2 cm segment, whereas subject F7, who received 26-week hydrochlorothiazide, showed concentrations ranging from 42 to 162 pg/mg in segments 0 to 8 cm.

**Table 1:**
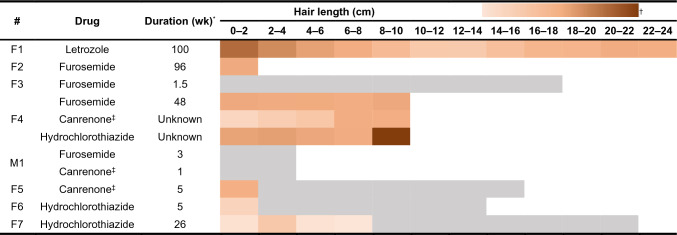
Drug distribution in hair samples obtained from subjects receiving diuretics and masking agents

F1: Female; Femara^®^ 2.5 mg QD; Perm & Dye

F2: Female; Uretropic^®^ 20 mg QD

F3: Female; Rasitol^®^ 40 mg Q12H

F4: Female; Rasitol^®^ 40 mg Q8H, Losa & Hydro^®^ 50/12.5 mg QD, spironolactone 25 mg BID

M1: Male; Rasitol^®^ 20 mg QD, Aldactone^®^ 25 mg QD

F5: Female; Aldactone^®^ 25 mg QD

F6: Female; Co-diovan FC^®^ 80/12.5 mg QD; Perm & Dye

F7: Female; Sevikar HCT^®^ 40/5/12.5 mg QD; Dye

^*^The duration of drug use prior to hair collection

^†^The colour scale represents the estimated drug concentration in hair segments, ranging 50–2600 pg/mg. Grey indicates not detected

^‡^The patients took spironolactone, and their hair was analyzed for canrenone, an active metabolite of spironolactone

## Discussion

In doping control, analyzing biological samples is crucial for identifying the presence or absence of a prohibited substance in the body. This study delved into comparing the presence of multiple compounds among conventional and alternative biological matrices, with a particular emphasis on exploring the analytical implications for hair samples.

Each of the biological samples has its own set of advantages and disadvantages. The preferred matrix for quantitative analysis of a substance to determine the extent of exposure is blood, as its concentration shows the strongest correlation with pharmacological responses (Quraishi et al. 2017). The dose-dependent effects were well depicted in our data. However, in routine testing, blood offers a very limited detection window, and its collection is deemed invasive, while the need for cold shipping conditions adds to the logistical challenges. Urine's non-invasiveness and good traceability for small molecules has taken a preeminent role in the screening of the majority of drugs. The parent form is biotransformed into excreted metabolites that subsequently manifest in the urine. This also explains the lack of detection of the parent substance in the urine in our results. The primary urinary metabolites for identifying stanozolol administration are 3-OH-stanozolol and 16β-OH-stanozolol (Delcourt et al. 2021; Schänzer et al. 1990). Tamoxifen is biotransformed to a large extent into 4-OH-tamoxifen and 4-OH-N-desmethyltamoxifen (Kisanga et al. 2005; Mihailescu et al. 2000), clomifene is converted to 4-OH-clomifene (Euler et al. 2022), and GW1516 is metabolised to GW1516-sulfoxide and GW1516-sulfone (Thevis et al. 2010).

The present study has demonstrated that hair detectability for chronic use of substances prohibited in sports outweighs that of other matrices. Some publications indicate the existence of compounds from each WADA class in hair samples. Anabolic steroids, being highly lipophilic molecules, are well-identified in human hair. For example, stanozolol, boldenone, trenbolone, metandienone, clostebol, drostanolone, metandienone, 19-norandrostenedione, and testosterone esters have been detected (Deshmukh et al. 2010; Gheddar et al. 2020; Kintz et al. 1999, 2021a, b) and even some emerging selective androgen receptor modulators (Kintz et al. 2022). This phenomenon aligns with our prediction model that most anabolic agents have a high likelihood of being incorporated into hair (Fig. 7). Peptide hormones and growth factors as large molecules cannot undergo passive diffusion from the blood capillaries to reach the hair follicle. The largest drug detected in hair is possibly cyclosporin A (molecular weight 1202.6 Da), a cyclic polypeptide immunosuppressant (Müller et al. 2013). Among hormone and metabolic modulators, the presence of the aromatase inhibitor letrozole and the PPARδ agonist GW1516 has been confirmed in human hair (Favretto et al. 2019; Kintz et al. 2020a). Studies on beta-2 agonists in hair are relatively limited. Clenbuterol has been reported (currently listed under the class of anabolic agents) (Kintz et al. 2019; Krumbholz et al. 2014) and identified in our study; however, terbutaline and salbutamol were not detected. Several studies, mostly in the context of examining feed additives in livestock feeding, described low but quantifiable salbutamol in hair, where salbutamol was administered for 21–28 days (see Table S6 for a summary). Vulić et al. administered 2.5 mg/kg of salbutamol to mice for 28 days and found 23.9 ng/g and 16.4 ng/g in black and white hair samples, respectively (Vulić et al. 2011). Other studies focused on large animals such as pigs, sheep, and cattle; hence, the treatment doses of salbutamol that are not suitable for comparison with the current findings (Chang et al. 2018; Decheng et al. 2015; Liu et al. 2019). Therefore, we tentatively conclude that detecting beta-2 agonists in hair requires a longer treatment duration than our study or possibly a larger dose. Our findings confirm that the non-detection of beta-2 agonists in hair does not necessarily imply a lack of usage.

Diuretics are abused by athletes in sports with weight classes, such as weightlifting, boxing, wrestling, and judo, to excrete water for rapid weight loss. Diuretics can also mask the presence of other banned substances, and they are thus prohibited both in competition and out of competition by the WADA (Cadwallader et al. 2010). The previous understanding regarding diuretics and masking agents suggests that their incorporation into hair is limited due to their acidic chemical characteristics, posing difficulties in detecting them (Kintz et al. 2020b). Interestingly, our study revealed that diuretics exhibit the highest level of incorporation among all classes of substances, indicating that diuretics are readily deposited in hair through the bloodstream. This has further piqued our interest in conducting the proof-of-concept human study. The detectability of common choices of diuretics, furosemide, hydrochlorothiazide, and canrenone, was then evidenced. From subject F1, we can conclude that dyeing, perming, exposure to UV, and shampooing can markedly decrease the drug concentrations, but they still remain present throughout the entire strand of hair and maintain consistent levels in the middle and distal segments. However, hair testing is not effective in determining drug use within the month preceding collection. Although hair should be collected as close to the scalp as possible, the follicles are typically located 5 mm beneath the scalp, and the hair with incorporated drugs only emerges from the follicle 2–3 weeks following drug administration (Joseph Jr et al. 1999).

Canrenone is an active metabolite of spironolactone, which is the most widely used steroidal antimineralocorticoid. Spironolactone is used in the treatment of heart failure as a 'potassium-sparing diuretic' and is also frequently prescribed to treat acne and excessive hair growth in women. The presence of canrenone in human hair is also a novel finding that has not been reported elsewhere.

## Conclusions

This study systematically evaluated the detectability of prohibited substances in sports through a multifaceted approach. It encompassed the administration of 17 model drugs to animals, in-silico prediction for over 200 drugs, and subsequent human investigation, specifically focusing on diuretic use. When comparing the detectable substances across different matrices, plasma demonstrated optimal dose dependencies, albeit with limited detection windows. Urine, faeces, and hair exhibited a coherent relationship with the administered dose for the majority of substances.

Significantly, hair manifested the highest probability of detection (14 out of 17) for selected compounds, encompassing anabolic agents, hormone and metabolic modulators, and diuretics, in contrast to other matrices. Within each substance class, beta-2 agonists were notably absent in hair, while diuretics and masking agents exhibited the highest degree of incorporation. The detectability of diuretics was robustly confirmed through the analysis of authentic human hair, and the temporal extent of their use was delineated through segmental analysis. Anabolic agents were predicted to exhibit a high level of confidence in their presence in hair. Conversely, undetectable compounds were inferred to primarily pertain to peptide hormones, growth factors, and beta-2 agonists, potentially attributed to their large molecular mass or high polarity.

Upon identifying the detectable compounds in hair, hair is considered a promising specimen to complement current doping control, particularly in the result management process. Segmented hair testing allows for the estimation of the timeframe of substance use, enabling the distinction between deliberate abuse and inadvertent exposure. Despite these advantages, several limitations in hair testing are acknowledged. Firstly, in practical scenarios, hair testing may not offer strong evidence for substances that need quantification. It is not suitable for identifying specific compounds that adhere to reporting thresholds. These compounds only raise concerns when their concentrations surpass designated cut-off levels, designed to exclude results from the use of a permitted source or dosage route. Despite finding satisfactory dose dependencies for most compounds in this controlled animal experiments, drug concentrations in actual human hair can be significantly influenced by various environmental factors, including UV exposure, shampooing, dyeing, and other external conditions. Secondly, drug stability in hair is seldom discussed and remains to be explored. A future comprehensive evaluation of the storage conditions of hair is warranted for doping control purposes.

### Supplementary Information

Below is the link to the electronic supplementary material.

Supplementary file1 (DOCX 234 kb)

Supplementary file2 (XLSX 27 kb)

## Data Availability

The datasets generated during and/or analyzed during the current study are available from the corresponding author on reasonable request.
