# Supplementary material for: Probing the hair detectability of prohibited substances in sports: an in vivo-in silico-clinical approach and analytical implications compared with plasma, urine, and faeces
Source: Arch Toxicol. 2024 Jan 15;98(3):779–90. doi: 10.1007/s00204-023-03667-1 (PMC10861659; doi:10.1007/s00204-023-03667-1)
Supplement: Supplementary file 1 — Supplementary file1 (DOCX 234 kb) [file 204_2023_3667_MOESM1_ESM.docx]

***Supplementary Information***

**Probing the** **hair detectability of prohibited substances in sports: An *in vivo*-*in silico*-clinical approach and analytical implications compared with plasma, urine, and faeces**

Shao-Hsin Hung ^a^, Hung-Lin Kan ^b^, Chun-Wei Tung ^b^, Yi-Ching Lin ^a,c^, Ting-Ting Chen ^d^, Ciao Tian ^e^, William Chih-Wei Chang ^a,e*^

1. Doctoral Degree Program in Toxicology, College of Pharmacy, Kaohsiung Medical University, Kaohsiung 807, Taiwan
2. Institute of Biotechnology and Pharmaceutical Research, National Health Research Institutes, Miaoli County 350, Taiwan
3. Department of Laboratory Medicine, School of Medicine, College of Medicine, Kaohsiung Medical University, Kaohsiung 807, Taiwan
4. Department of Leisure Industry and Health Promotion, College of Humanities and Management, National Ilan University, Ilan County 260, Taiwan
5. School of Pharmacy, College of Pharmacy, Kaohsiung Medical University, Kaohsiung 807, Taiwan

*Corresponding author

William Chih-Wei Chang, PhD

100, Shih-Chuan 1st Rd., Sanmin Dist., Kaohsiung 807, Taiwan

Telephone: 886-7-312-1101 (ext. 2653)

E-mail: [cwchang@kmu.edu.tw](mailto:cwchang@kmu.edu.tw)

**Text S1. Standards, chemicals, and reagents**

The reference standard clomifene citrate was purchased from AK Scientific (Union City, CA, USA); terbutaline was from Cayman Chemical Company (Ann Arbor, MI, USA); salbutamol, tamoxifen, GW1516, trimetazidine, and canrenone were from Combi-Blocks (San Diego, CA, US); stanozolol, methyltestosterone, testosterone, clenbuterol hydrochloride, anastrozole, letrozole, furosemide, hydrochlorothiazide, chlorothiazide, and probenecid were from Tokyo Chemical Industry (Tokyo, Japan). The stable isotope labelled standard (used as internal standard) testosterone-d3 was purchased from Cerilliant (Austin, TX, US); clenbuterol-d9, terbutaline-d9, tamoxifen-d5, and furosemide-d5 were from Toronto Research Chemicals (North York, ON, Canada).

All the solvents and reagents used in this study were of LC or LC−MS grade. Methanol was obtained from J.T. Baker (Phillipsburg, NJ, USA), acetonitrile was from Duksan (Ansan, South Korea), formic acid was from Honeywell Research Chemicals (Seelze, Germany), and buffer solution pH 7.00 (di-sodium hydrogen phosphate/potassium dihydrogen phosphate) was from Merck (Rahway, NJ, USA). Pure water was prepared using the Milli-Q system (Millipore, Bedford, MA, USA).

Corn oil was acquired from Sigma-Aldrich (St. Louis, MO, USA). β-Glucuronidase from Escherichia coli (E. coli) K 12, used for the hydrolysis of the glucuronide-conjugated drugs, was from Roche Diagnostic (Mannheim, Germany).

**Text S2. Preliminary optimisation for hair sample preparation**

**Decontamination**

While only the exposed substances that enter the body and incorporate into the hair shaft are of interest, the decontamination of a hair sample is a mandatory step to eliminate external contaminants. Typically, the decontamination procedure consists of two washing steps with both organic solvent and aqueous solution ^1^. Protic solvents such as methanol will swell the hair and possibly extract drugs from the inner part of the hair ^2^. Non-protic solvents such as dichloromethane will ideally remove only the surface contamination. Therefore, we chose dichloromethane and pure water as the washing solvents.

**Homogenisation**

Since the hair shaft is made of hard keratinised cells, sufficient homogenisation may improve the release of the incorporated analytes. Comparative experiments were conducted to investigate the homogenisation (a) w or w/o bead-beating, and bead-beating conditions (b) w or w/o buffer, (c) types of beads, (d) sizes of beads, and (e) run time. First, hair was cut into snippets, then pulverised by the bead-beating method using a bullet blender (Next Advance Co., Averill Park, NY, USA). By comparing methods between (a) w or w/o bead-beating, significant enhancements of intensities were found in clenbuterol (α = 0.006), terbutaline (α = 0.048), clomifene (α = 0.002), anastrozole (α = 0.018), trimetazidine (α = 0.025), hydrochlorothiazide (α = 0.013), chlorothiazide (α = 0.002), and probenecid (α = 0.035).

**
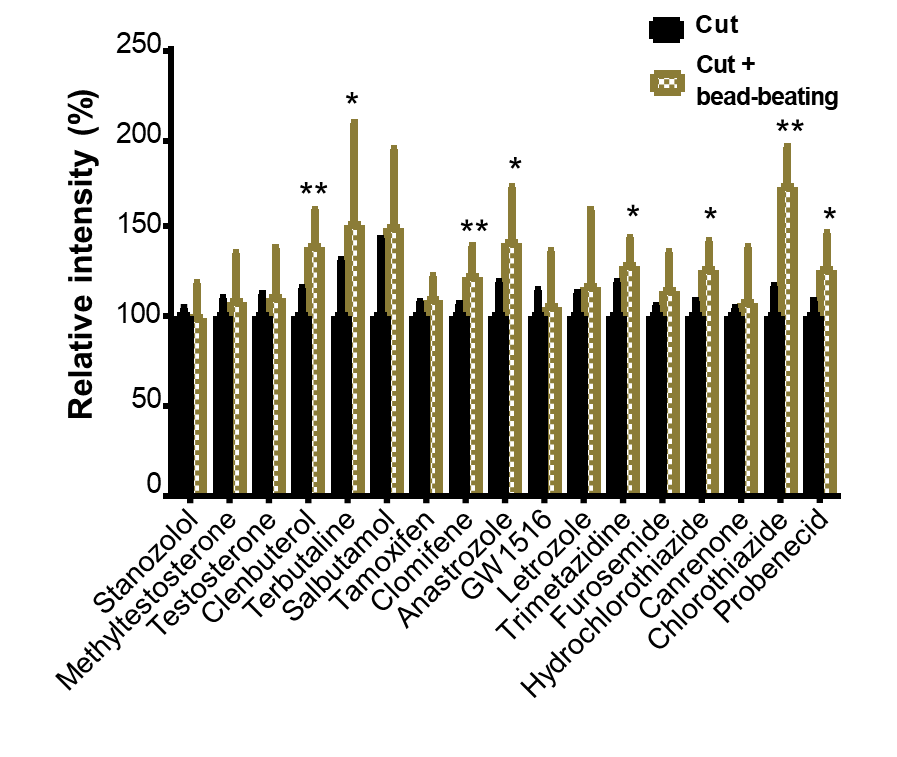
**

Note: Effects of w or w/o bead-beating during hair homogenisation on relative intensities of the analytes. Real hair samples after rat oral gavage were used for the comparison. Data (*n* = 7) are presented as mean ± standard deviation. Mann-Whitney U test was used for determining the differences between the two groups. Statistical significances are denoted as * α < 0.050 and ** α < 0.010.

Regarding (b) w or w/o buffer, no addition of buffer gives better results because the samples are rapidly agitated with grinding media (beads) and buffer can act as a cushion during the impact. Regarding (c) types of beads, the stainless-steel bead gives better results. The samples are not homogenised successfully and are still in clumps using the ZrO bead. This is probably because hair as a hard matrix requires a higher density of beads to disrupt it. The stainless-steel bead is denser than the ZrO bead. Regarding (d) sizes of beads, the mixed 0.9–2.0 mm beads give better results. The 1.6 mm beads are too large in 1.5-mL microtubes and not able to produce enough impact within the space. Regarding (e) run time, a 10-min operation gives better results than 3-min but no apparent temperature rise. As a result, the homogenisation procedure includes cutting into snippets and then pulverising with stainless steel beads 0.9–2.0 mm using a bullet blender operated for 10 min.

| With buffer  **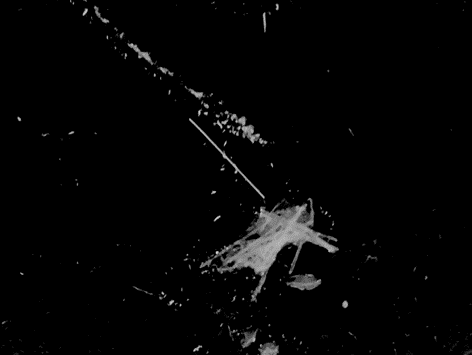** | **Without buffer**  **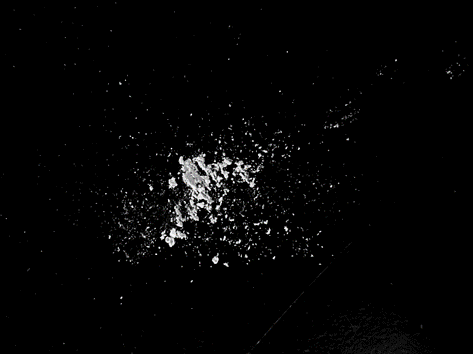** |
| --- | --- |
| ZrO bead  **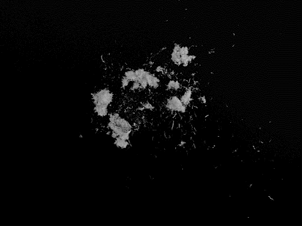** | **Stainless steel bead**  **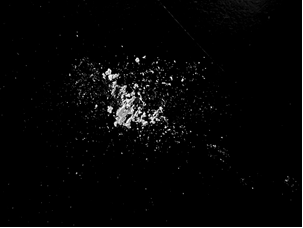** |
| **0.9–2.0 mm bead**  **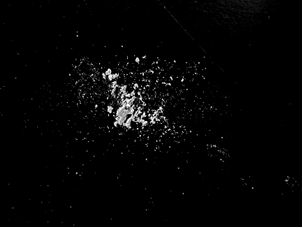** | 1.6 mm bead  **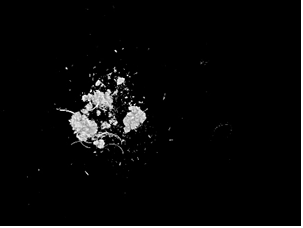** |
| 3 min runtime  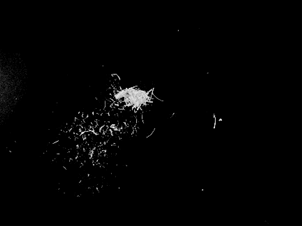 | **10 min** **runtime**  **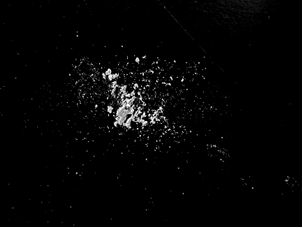** |

Note: the conditions of bead-beating with better performance are presented in bold text.

**Text S3. Sample preparation for hair, plasma, urine, and faeces**

**Hair:** Ten-milligram of hair samples were firstly washed with dichloromethane (1 mL), then the liquid was discarded, and the samples were dried under nitrogen. The samples were secondly washed with pure water (1 mL), then the liquid was discarded again, and the samples were dried overnight in the oven at 25°C. The dried samples were cut into snippets then pulverised thoroughly with stainless steel beads 0.9–2.0 mm using a bullet blender (Next Advance Co., Averill Park, NY, USA) operated at power setting 10 for 10 min. Afterwards, the samples were mixed with 10 µL of deuterated internal standards (1,500 ng/mL), 50 µL of pure water, and 240 µL of acetonitrile and incubated at 1,500 rpm (50 °C) for 4 h. The final extract of 300 µL was centrifuged at 3,000 rpm for 10 min. The supernatants were transferred to a new tube and centrifuged again at 13,000 rpm for 10 min. The supernatants were collected for analysis.

**Plasma:** Thirty-microlitre of plasma samples were mixed with 10 µL of deuterated internal standards (1,500 ng/mL) and 260 µL of acetonitrile and incubated at 1,500 rpm for 10 min. The final extract of 300 µL was centrifuged at 13,000 rpm for 10 min and the supernatants were collected for analysis.

**Urine:** Fifty-microlitre of urine samples were mixed with 50 μL of phosphate buffer (pH 7.00), 10 μL of β-glucuronidase, and incubated at 50 °C for 1 h. Then, 180 µL of acetonitrile and 10 µL of deuterated internal standards (1,500 ng/mL) were added and incubated at 1,500 rpm for 10 min. The final extract of 300 µL was centrifuged at 13,000 rpm for 10 min and the supernatants were collected for analysis.

**Faeces:** Ten-milligram of faecal samples were mixed with 10 µL of deuterated internal standards (1,500 ng/mL), 50 µL of pure water, and 240 µL of acetonitrile and incubated at 1,500 rpm for 10 min. The final extract of 300 µL was centrifuged at 13,000 rpm for 10 min and the supernatants were collected for analysis.

**Table S1. AAF occurrence rate, logP, and LD_50_ of the model drugs**

| **Class** | **Compound** | **AAF occurrence rate**  **(% within drug class)** | **LogP** | **LD_50_** |
| --- | --- | --- | --- | --- |
| **S1.1 Anabolic Agents** | Stanozolol | 18% | 4.42 | 980 mg·kg^–1^ rat |
|  | Methyltestosterone | 1% | 3.69 | 2,500 mg·kg^–1^ rat |
|  | Testosterone | 0.3% | 3.25 | >500 mg·kg^–1^ rat |
| **S1.2 Other Anabolic Agents** | Clenbuterol | 56% | 2.79 | 159 mg·kg^–1^ rat |
| **S.3 Beta-2 Agonists** | Terbutaline | 52% | 1.07 | 1,800 mg·kg^–1^ rat |
|  | Salbutamol | 7% | 1.35 | 660 mg·kg^–1^ rat |
| **S.4 Hormone and Metabolic Modulators** | Tamoxifen | 22% | 6.06 | 4,100 mg·kg^–1^ rat |
|  | Clomifene | 20% | 6.53 | 5,750 mg·kg^–1^ rat |
|  | Anastrozole | 10% | 2.85 | >100 mg·kg^–1^ rat |
|  | GW1516 | 9% | 5.85 | 980 mg·kg^–1^ rat |
|  | Letrozole | 8% | 2.15 | >2,000 mg·kg^–1^ rat |
|  | Trimetazidine | 1% | 0.87 | 1,700 mg·kg^–1^ rat |
| **S.5 Diuretics and Other Masking Agents** | Furosemide | 29% | 1.77 | 2,600 mg·kg^–1^ rat |
|  | Hydrochlorothiazide | 21% | -0.06 | >2,750 mg·kg^–1^ rat |
|  | Canrenone | 10% | 2.03 | >5,000 mg·kg^–1^ rat |
|  | Chlorothiazide | 7% | 0.02 | >10,000 mg·kg^–1^ rat |
|  | Probenecid | 4% | 2.92 | 1,600 mg·kg^–1^ rat |

Occurrences of Adverse Analytical Findings (AAFs) - Substances Identified as AAFs in Each Drug Class

LogP was calculated using Molinspiration logP predictor ^3^.

References

(1) Cooper, G. A.; Kronstrand, R.; Kintz, P. Society of Hair Testing guidelines for drug testing in hair. *Forensic Science International* **2012**, *218* (1-3), 20-24.

(2) Vogliardi, S.; Tucci, M.; Stocchero, G.; Ferrara, S. D.; Favretto, D. Sample preparation methods for determination of drugs of abuse in hair samples: a review. *Analytica Chimica Acta* **2015**, *857*, 1-27.

(3) Slovensky Grob. *Molinspiration Cheminformatics free web services*. 2022. <https://www.molinspiration.com> (accessed 18 Oct 2022).

| **Table S2. Optimised mass spectrometric settings for each analyte** | | | | | | |
| --- | --- | --- | --- | --- | --- | --- |
| **Class** | **Compound** | **RT** | **Formula** | **MRM transitions** | **Cone voltage (V)** | **Collision energy (eV)** |
| S1.1 Anabolic agents | Stanozolol | 5.47 | C_21_H_32_N_2_O | 329.3→81  329.3→95  329.3→121 | 60  80  60 | 44  38  34 |
|  | Methyltestosterone | 6.00 | C_20_H_30_O_2_ | 303.2→97  303.2→109  303.2→107 | 35  75  35 | 22  30  22 |
|  | Testosterone | 5.67 | C_19_H_28_O_2_ | 289.2→97  289.2→109  289.2→79 | 30  30  8 | 21  25  54 |
|  | Testosterone-*d*3 | 5.66 | C_19_H_25_D_3_O_2_ | 292.2→97  292.2→109  292.2→79 | 30  30  8 | 21  25  54 |
| S1.2 Other anabolic agents | Clenbuterol | 2.78 | C_12_H_18_Cl_2_N_2_O | 277.1→203  277.1→168  277.1→57 | 25  20  40 | 22  30  32 |
|  | Clenbuterol-*d*9 | 2.77 | C_12_H_9_D_9_C_l2_N_2_O | 286.1→203  286.1→168  286.1→57 | 25  20  40 | 22  30  32 |
| S.3 Beta-2 agonists | Terbutaline | 1.21 | C_12_H_19_NO_3_ | 226.1→152  226.1→125  226.1→107 | 25  25  82 | 22  33  30 |
|  | Salbutamol | 1.26 | C_13_H_21_NO_3_ | 240.2→148  240.2→133  240.2→166 | 74  40  74 | 16  29  10 |
|  | Terbutaline-*d*9 | 1.21 | C_12_H_10_D_9_NO_3_ | 235.2→152  235.2→125  235.2→107 | 25  25  82 | 22  33  30 |
| S.4 Hormone and metabolic modulators | Tamoxifen | 6.78 | C_26_H_29_NO | 372.2→72  372.2→57  372.2→128 | 34  34  74 | 26  56  53 |
|  | Clomiphene | 6.52 | C_26_H_28_ClNO | 406.2→100  406.2→72  406.2→58 | 82  82  82 | 26  36  42 |
|  | Anastrozole | 4.63 | C_17_H_19_N_5_ | 294.2→225  294.2→142  294.2→116 | 41  38  38 | 16  54  56 |
|  | GW1516 | 8.41 | C_21_H_18_F_3_NO_3_S_2_ | 454.1→257  454.1→188  454.1→85 | 91  91  2 | 32  48  66 |
|  | Letrozole | 4.57 | C_17_H_11_N_5_ | 286.1→217  286.1→190 | 44  44 | 12  32 |
|  | Trimetazidine | 1.38 | C_14_H_22_N_2_O_3_ | 267.2→181  267.2→166  267.2→91 | 26  26  26 | 42  24  12 |
|  | Tamoxifen-*d*5 | 6.79 | C_26_H_24_D_5_NO | 377.3→72  377.3→57  377.3→128 | 34  34  74 | 26  56  53 |
| S.5 Diuretics and other masking agents | Furosemide | 4.33 | C_12_H_11_ClN_2_O_5_S | 329.0→285  329.0→205  329.0→78 | 40  46  46 | 13  20  30 |
|  | Hydrochlorothiazide | 1.75 | C_7_H_8_ClN_3_O_4_S_2_ | 296.0→78  296.0→269  296.0→205 | 64  64  64 | 32  16  20 |
|  | Canrenone | 6.19 | C_22_H_28_O_3_ | 341.2→107  341.2→73  341.2→91 | 100  100  140 | 28  30  30 |
|  | Chlorothiazide | 1.59 | C_7_H_6_ClN_3_O_4_S_2_ | 293.9→179  293.9→214 | 4  4 | 50  50 |
|  | Probenecid | 5.95 | C_13_H_19_NO_4_S | 284.1→140  284.1→240  284.1→76 | 50  50  4 | 20  20  36 |
|  | Furosemide-*d*5 | 4.30 | C_12_H_11_ClN_2_O_5_S | 334.0→78  334.0→205  334.0→285 | 46  46  40 | 30  20  13 |

| **Table S3. Linear ranges for semi-quantitation of each analyte between hair, plasma, urine, and faeces** | | | | | | | | | |
| --- | --- | --- | --- | --- | --- | --- | --- | --- | --- |
| **Class** | **Compound** | **Hair** | | **Plasma** | | **Urine** | | **Faeces** | |
|  |  | **Linear range (pg/mg)** | **r^2^** | **Linear range (ng/mL)** | **r^2^** | **Linear range (ng/mL)** | **r^2^** | **Linear range (pg/mg)** | **r^2^** |
| S1.1 Anabolic agents | Stanozolol | 0.01 – 500 | 0.993 | 0.01 – 500 | 0.997 | 0.01 – 500 | 0.997 | 0.01 – 500 | 0.992 |
|  | Methyltestosterone | 0.025 – 500 | 0.988 | 0.01 – 500 | 0.994 | 0.01 – 500 | 0.997 | 0.05 – 500 | 0.992 |
|  | Testosterone | 0.025 – 500 | 0.992 | 0.01 – 500 | 0.99 | 0.01 – 500 | 0.998 | 0.025 – 500 | 0.993 |
| S1.2 Other anabolic agents | Clenbuterol | 0.01 – 500 | 0.988 | 0.01 – 500 | 0.998 | 0.01 – 500 | 0.999 | 0.01 – 500 | 0.993 |
| S.3 Beta-2 agonists | Terbutaline | 0.25 – 500 | 0.991 | 0.25 – 500 | 0.996 | 0.25 – 500 | 0.997 | 1 – 500 | 0.991 |
|  | Salbutamol | 0.1 – 500 | 0.987 | 0.5 – 500 | 0.994 | 5 – 500 | 0.991 | 10 – 500 | 0.993 |
| S.4 Hormone and metabolic modulators | Tamoxifen | 0.01 – 500 | 0.997 | 0.01 – 500 | 0.998 | 0.01 – 500 | 0.993 | 0.025 – 500 | 0.997 |
|  | Clomiphene | 0.01 – 500 | 0.994 | 0.01 – 500 | 0.996 | 0.01 – 500 | 0.996 | 0.05 – 500 | 0.993 |
|  | Anastrozole | 0.01 – 500 | 0.993 | 0.01 – 500 | 0.997 | 0.01 – 500 | 0.999 | 0.01 – 500 | 0.991 |
|  | GW1516 | 0.01 – 500 | 0.991 | 0.01 – 500 | 0.996 | 0.01 – 500 | 0.999 | 0.025 – 500 | 0.990 |
|  | Letrozole | 0.01 – 500 | 0.991 | 0.01 – 500 | 0.993 | 0.01 – 500 | 0.996 | 0.025 – 500 | 0.991 |
|  | Trimetazidine | 2.5 – 500 | 0.993 | 0.1 – 500 | 0.995 | 0.01 – 500 | 0.992 | 1 – 500 | 0.994 |
| S.5 Diuretics and other masking agents | Furosemide | 10 – 500 | 0.993 | 0.1 – 500 | 0.997 | 0.01 – 500 | 0.99 | 1 – 500 | 0.993 |
|  | Hydrochlorothiazide | 10 – 500 | 0.988 | 5 – 500 | 0.993 | 1 – 500 | 0.994 | 10 – 500 | 0.995 |
|  | Canrenone | 0.5 – 500 | 0.991 | 0.25 – 500 | 0.996 | 0.25 – 500 | 0.995 | 1 – 500 | 0.990 |
|  | Chlorothiazide | 1 – 500 | 0.983 | 5 – 500 | 0.993 | 2.5 – 500 | 0.991 | 10 – 500 | 0.997 |
|  | Probenecid | 0.025 – 500 | 0.991 | 0.025 – 500 | 0.997 | 0.01 – 500 | 0.993 | 0.1 – 500 | 0.993 |

| **Table S4. The concentrations of each analyte in four specimens among three dosage groups** | | | | | | | | | | | | | | | |
| --- | --- | --- | --- | --- | --- | --- | --- | --- | --- | --- | --- | --- | --- | --- | --- |
| **Compound** | **Hair conc.** | | | **Plasma C_max_** | | | **Plasma AUC** | | | **Urine C_max_** | | | **Faeces C_max_** | | |
|  | **0.5X** | **1X** | **2X** | **0.5X** | **1X** | **2X** | **0.5X** | **1X** | **2X** | **0.5X** | **1X** | **2X** | **0.5X** | **1X** | **2X** |
| Stanozolol | 8.17 (1.23) | 8.05 (1.62) | 20.81 (1.19) | 3.39 (1.02) | 10.09 (4.32) | 19.67 (5.95) | 24.77 (6.36) | 72.78 (23.77) | 156.4 (39.50) | ND | ND | ND | 136.8 (109.7) | 214.1 (120.3) | 1015.9 (668.5) |
| Methyltestosterone | 137.4 (6.11) | 141.6 (6.36) | 48.89 (1.95) | ND | ND | ND | ND | ND | ND | ND | ND | ND | ND | ND | ND |
| Testosterone | 45.15 (4.24) | 30.66 (1.91) | 75.07 (3.56) | ND | ND | ND | ND | ND | ND | ND | ND | ND | ND | ND | ND |
| Clenbuterol | 69.30 (10.89) | 196.6 (57.85) | 1129.3 (160.4) | 72.76 (10.36) | 258.1 (34.92) | 286.7 (38.36) | 565.0 (76.80) | 6317.1 (802.6) | 7174.8 (1334.1) | 1362.5 (237.0) | 2530.3 (486.0) | 2349.4 (465.5) | 132.6 (76.06) | 306.2 (157.2) | 941.4 (614.4) |
| Terbutaline | ND | ND | ND | ND | ND | ND | ND | ND | ND | 1188.0 (168.5) | 1963.0 (267.6) | 1251.5 (188.4) | 6066.0 (3180.6) | 6881.6 (2623.5) | 12190 (7372.6) |
| Salbutamol | ND | ND | ND | ND | ND | ND | ND | ND | ND | 1615.2 (184.6) | 2706.6 (570.8) | 5720.1 (940.2) | 2145.1 (1036.2) | 3115.4 (1317.5) | 3591.7 (2027.6) |
| Tamoxifen | 25.87 (3.45) | 55.44 (12.78) | 210.6 (17.78) | 24.58 (1.40) | 65.87 (4.69) | 118.6 (9.33) | 220.5 (11.46) | 538.0 (55.10) | 3949.5 (354.8) | ND | ND | ND | 786.9 (391.8) | 1224.4 (837.3) | 5220.6 (2612.0) |
| Clomifene | 14.78 (2.33) | 27.72 (6.96) | 92.56 (8.99) | 27.98 (1.40) | 74.12 (5.23) | 161.5 (16.49) | 242.8 (11.93) | 599.5 (45.71) | 2152.4 (501.4) | ND | ND | ND | 840.5 (475.5) | 841.4 (591.2) | 5511.2 (3016.2) |
| Anastrozole | 143.8 (20.00) | 202.5 (22.25) | 411.2 (49.55) | 234.3 (17.01) | 522.8 (50.57) | 816.3 (294.3) | 2318.7 (108.0) | 4914.9 (377.2) | 10566 (1928.7) | 190.4 (43.76) | 331.7 (75.63) | 247.0 (44.11) | 115.2 (73.18) | 139.2 (113.5) | 1310.2 (1273.5) |
| GW1516 | 282.8 (52.24) | 421.2 (268.9) | 1387.6 (161.6) | 90.71 (11.88) | 149.2 (26.31) | 516.5 (136.5) | 3070.4 (347.5) | 4763.0 (886.3) | 13497 (1508.2) | ND | ND | ND | 7506.4 (2002.2) | 11870 (6149.3) | 13632 (5017.3) |
| Letrozole | 300.5 (51.59) | 409.6 (70.71) | 1040.3 (124.2) | 356.8 (21.21) | 679.2 (36.40) | 1049.7 (177.6) | 3540.4 (222.5) | 6781.5 (506.4) | 20401 (3507.2) | 206.4 (23.24) | 435.1 (72.27) | 403.9 (77.95) | 520.7 (179.1) | 806.6 (384.8) | 2225.1 (995.4) |
| Trimetazidine | 19.51 (4.51) | 39.53 (4.24) | 176.7 (24.00) | 19.79 (4.12) | 64.58 (20.82) | 79.00 (11.32) | 123.9 (11.41) | 425.7 (86.57) | 1258.2 (447.9) | 29017 (10183) | 56217 (22776) | 22307 (3890.1) | 293.2 (140.9) | 322.0 (216.1) | 628.9 (343.7) |
| Furosemide | 105.4 (15.07) | 161.9 (23.47) | 350.3 (54.81) | 47.49 (12.04) | 60.66 (10.45) | 299.8 (178.5) | 275.1 (36.41) | 518.5 (49.38) | 938.5 (184.3) | 1405.4 (259.0) | 2252.2 (587.3) | 2035.4 (260.7) | 6579.0 (3429.0) | 6728.0 (2228.8) | 15873 (8962.7) |
| Hydrochlorothiazide | 283.5 (38.85) | 222.3 (59.36) | 892.9 (96.52) | ND | ND | ND | ND | ND | ND | 1580.7 (158.4) | 2570.8 (427.9) | 2924.1 (143.4) | 4831.2 (385.0) | 6092.6 (2615.2) | 9355.1 (4962.2) |
| Canrenone | 57.13 (9.54) | 66.08 (13.27) | 450.1 (17.77) | ND | ND | ND | ND | ND | ND | ND | ND | ND | ND | ND | ND |
| Chlorothiazide | ND | ND | ND | ND | ND | ND | ND | ND | ND | 757.2 (105.9) | 1794.7 (264.0) | 1801.6 (122.8) | 10279 (4182.5) | 10667 (5502.3) | 20158 (10231) |
| Probenecid | 32.87 (3.66) | 41.78 (6.04) | 121.5 (8.48) | 7.37 (1.67) | 25.90 (12.54) | 78.74 (40.62) | 51.18 (7.97) | 120.4 (14.08) | 173.6 (24.50) | ND | ND | ND | ND | ND | ND |
| Note: Data present as mean (standard error of the mean); ND = not detected. | | | | | | | | | | | | | | | |

| **Table S6. Studies on the analysis of salbutamol in hair samples** | | | | |
| --- | --- | --- | --- | --- |
| **No.** | **Animal subject** | **Dose and duration of the treatment** | **Hair concentration** | **Reference** |
| 1 | Mouse | Salbutamol 2.5 mg/kg (28 days) | 16.4 – 23.9 ng/g | Vulić A, Pleadin J, Perši N, Stojković R, Ivanković S (2011) Accumulation of β-agonists clenbuterol and salbutamol in black and white mouse hair. *Journal of Analytical Toxicology* 35(8):566-570 |
| 2 | Pig | Salbutamol 5 ppm (28 days) | 0.5 ppm | Chang K-C, Chang Y-T, Tsai C-E (2018) Determination of ractopamine and salbutamol in pig hair by liquid chromatography tandem mass spectrometry. *Journal of Food and Drug Analysis* 26(2):725-730 |
| 3 | Cattle | Salbutamol 50.0 μg/kg (28 days) | 100.6 ng/g | Liu J, Tang C, Long R, et al. (2019) The use of hair as a long‐term indicator of low‐dose β2 agonist treatments in cattle: Implications for growth‐promoting purposes monitoring. *Drug Testing and Analysis* 11(6):745-751 |
| 4 | Sheep | Salbutamol 150 μg/kg (21 days) | 132 ng/g | Decheng S, Wei Z, Yu Z, et al. (2015) Validation of a confirmatory method of salbutamol in sheep hair by UPLC-MS/MS and its application to pharmacokinetic study. *Journal of Pharmaceutical and Biomedical Analysis* 114:12-15 |
